# Diversity and distribution of sodium channel mutations in *Aedes albopictus* (Diptera: Culicidae)

**DOI:** 10.1093/jme/tjae005

**Published:** 2024-02-16

**Authors:** Nancy M Endersby-Harshman, Thomas L Schmidt, Ary A Hoffmann

**Affiliations:** Pest and Environmental Adaptation Research Group, School of BioSciences, Bio21 Institute, The University of Melbourne, Parkville, Victoria, Australia; Pest and Environmental Adaptation Research Group, School of BioSciences, Bio21 Institute, The University of Melbourne, Parkville, Victoria, Australia; Pest and Environmental Adaptation Research Group, School of BioSciences, Bio21 Institute, The University of Melbourne, Parkville, Victoria, Australia

**Keywords:** target site, knockdown, invasion

## Abstract

There is growing interest in insecticide resistance in the mosquito, *Aedes albopictus* (Skuse), as its potential for spreading diseases is increasing as urbanization and control efforts intensify. Here we review the presence and diversity of mutations in the voltage-sensitive sodium channel (*Vssc*) gene associated with pyrethroid resistance and report on additional surveys of these mutations in new populations with an analysis of their spread. The known diversity of these mutations has increased in recent years including the identification of 26 non-synonymous mutations, although phenotypic data associating mutations with resistance remain limited. We provide data on mutations in several new locations including those in Timor Leste, Indonesia, and Vanuatu. We use population genomic data from ddRAD analyses of target populations with the 1534C mutation to identify single nucleotide polymorphisms (SNPs) associated with the mutant to test for clustering of SNPs based on the presence of the 1534C mutation rather than population origin. Our findings suggest spread of resistance alleles via genetic invasion, which is further supported by patterns from a genome-wide principal components analysis. These data point to movement of resistance alleles across wide areas with likely impacts on local control options.

## Introduction

Insecticide resistance in disease-vectoring mosquitoes is a significant problem for public health as it undermines vector control methods based on chemical applications. In particular, resistance to pyrethroid insecticides in vectors of dengue complicates emergency outbreak responses where rapid knockdown of mosquitoes within an infected area is required (Vazquez-Prokopec et al. 2017). The target site of both pyrethroid insecticides and dichlorodiphenyltrichloroethane (DDT) is the voltage-sensitive sodium channel (Vssc) protein which generates action potentials to produce a nerve impulse (Silver et al. 2014). Pyrethroid insecticides fall into 2 broad groups based on their chemical structure and poisoning symptoms (Davies et al. 2007). In susceptible insects, type I pyrethroids disrupt inactivated channels whereas type II pyrethroids bind selectively to open channels, fixing them in that state so that nervous system function in the insect is compromised (Silver et al. 2014). In resistant insects, mutations in the target site may cause reduced binding affinity of pyrethroids and possibly counteract the effects of pyrethroids on the gating function, meaning that some sodium channels can function normally and insects are not knocked down (Dong et al. 2014). Other mechanisms including detoxification of insecticides by metabolism may also result in pyrethroid resistance in *Aedes* mosquitoes (Bharati and Saha 2021).

Target-site mutations in the *Vssc* gene of the principal vector of dengue, *Aedes aegypti*, are widespread and well-studied (Smith et al. 2016). Until recently, *Vssc* mutations in *Aedes albopictus*, a secondary vector of dengue and a primary vector of chikungunya, have been reported only rarely and restricted to a single codon (1534) in domain III of the Vssc protein numbered according to the housefly genome (Kasai et al. 2011). The difference in *Vssc* mutation status of the 2 species has been attributed to differences in the degree to which *Ae*. *albopictus* comes into contact with humans and insecticides, compared with the anthropophilic *Ae*. *aegypti* (Vontas et al. 2012). However, as urbanization progresses, the distribution of *Ae*. *albopictus* increasingly overlaps that of human settlement bringing an increase in vectoring possibilities and insecticide exposure (Vontas et al. 2012, Zulfa et al. 2022).

A review published in 2012 (Vontas et al. 2012) noted that the only mutation in the *Vssc* gene of *Ae*. *albopictus* observed at that time was F1534C recorded from mosquitoes in Singapore (Kasai et al. 2011). *Aedes albopictus* remained susceptible to pyrethroid insecticides across a wide geographic area (Africa, Asia, and Italy) (Vontas et al. 2012), but some resistance to DDT had been noted in mosquitoes from Sri Lanka and Cameroon (Kamgang et al. 2011). Twelve years later, 26 non-synonymous mutations have now been reported within 12 codons in the *Vssc* gene of *Ae*. *albopictus* from 26 countries throughout Asia, the Indo-Pacific region, Europe, and the United States of America (Table 1). Furthermore, resistance in populations of *Ae*. *albopictus* has become more commonly reported, with studies from Asia showing incidence of resistance increasing significantly since 2010 (Zulfa et al. 2022). Resistance levels and frequency in *Ae*. *albopictus* still lag behind those found in *Ae*. *aegypti*, but indicate a concerning trend that should be addressed.

**Table 1.** *Aedes albopictus* worldwide distribution of *Vssc* mutations from the literature to date (2011–2023)

Codon	Wild-type codon	Laos	Thailand	Mexico	Italy	Vietnam	La Réunion	China	Greece	Albania	Bulgaria, France, Georgia, Malta, Turkey, Switzerland, Spain, Romania (GGA)	USA	Timor Leste	PNG, Singapore, Vanuatu	India	Malaysia & Indonesia
V410L	GTA		CTA*	CTA*	CTA*											
V410A	GTA				GCA											
V410G	GTA				GGG*											
S989P	TCC		CCC*													
S989Y	TCC			TAC*	TAC*											
S1000Y	TCC							TAC*								
I1011V	ATA				GTA*											
V1016G	GTA^c^				GGA	GGA	GGA*	GGA			GGA*				GGA*	
V1016I	GTA				ATA											
T1520I	ACC	ATC*													ATC	
I1532T	ATC^a^				ACC			ACC	ACC	ACC						
F1534C_1	TTC^b^					TGC		TGC	TGC				TGC*	TGC	TGC	
F1534C_2	TTC							TGT								
F1534C_1/F1534S_1	TTC							TGC/TCC								
F1534S/F1543I	TTC							ATC*								
F1534L_1	TTC			TTG	TTG			TTG								TTG
F1534L_2	TTC							CTC				CTC				
F1534L_3	TTC							TTA								
F1534L_4	TTC							CTG								
F1534L_1/F1534S_1	TTC							TTG/TCC								
F1534L_1/F1534S_2	TTC							TTG/TCG								
F1534R	TTC							CGC								
F1534S_1	TTC					?		TCC				TCC				
F1534S_2	TTC							TCG								
F1534S_1/F1534S_2	TTC							TCC/TCG								
F1534W	TTC							TGG								
D1763Y	GAC							TAC								

Red text indicates more than one mutation required.

Bule text indicates sequence deduced if only a single mutation occurs (because sequence not specified in reference).

*Heterozygote only.

^a^ATA also recorded as wild type by Zhou et al. (2019) in Beijing, China.

^b^TTT also recorded as wild type by Chen et al. (2016) in Hainan Island, China and by Yuan et al. (2023) in Shanghai, China.

^c^GTG also recorded as wild type by Pichler et al. (2021) and Zheng et al. (2022). Novel mutations C749 (Japan), A2023T (Singapore, Vietnam, and Japan), and G2046E (Japan) found in *Ae*. *albopictus* using exon capture (Itokawa et al. 2019) have not been included due to space restrictions.

*Aedes aegypti* and *Ae*. *albopictus* both move only short distances in their home range (Honorio et al. 2003, Jasper et al. 2019), but become highly mobile on occasion due to their affinity with human-mediated transport (Schmidt et al. 2020). The colonization of Europe and the United States of America by *Ae*. *albopictus,* which has an origin in Asia, is a prime example of this mobility (Benedict et al. 2007). In *Ae*. *aegypti,* the present geographic distribution of *Vssc* mutations in populations from the Indo-Pacific region has largely been produced by long-distance movement of mosquitoes carrying these mutations, rather than by selection on independent mutation events in each population (Endersby-Harshman et al. 2020). A question arises as to whether the same phenomenon is directing development of resistance in populations of *Ae*. *albopictus*.

Our study aimed to

- summarize published literature on *Vssc* mutants in *Ae*. *albopictus* by geographic location, codons affected, and specific point mutations;
- scrutinize population genomic SNP data in *Ae*. *albopictus* to look for signatures of selection to see if the *Vssc* gene is a site of interest; and
- compare *Vssc* sequence data with population genomic data to assess whether mutations in mosquito populations from particular geographic locations might have arisen by invasion of resistant genotypes.

## Methods

### Literature Review

Literature was surveyed up to June 2023 covering publications from 2016 to 2023 using the following search terms in the Discovery search function available through the University of Melbourne’s library: *Ae. albopictus*, *kdr*, mutation, sodium channel. The Discovery search engine looks at an extensive range of databases accessed through the University of Melbourne (https://unimelb.libguides.com/az.php). Additional searches using Google Scholar and ad hoc discoveries of references were also included with the aim of finding every published reference to *Vssc* mutations in *Ae*. *albopictus*. References were divided into 2 categories: those describing sodium channel mutations discovered in the *Vssc* gene of *Ae*. *albopictus* and those in which mutations were sought but not discovered.

### Additional *Vssc* Screening

Samples of *Ae*. *albopictus* from throughout the Indo-Pacific region were collected by multiple collaborators and stored in absolute ethanol. Samples were used initially to create a genomic reference databank for 2 studies using ddRADSeq (Schmidt et al. 2020, 2021). Sanger sequencing of 4 regions of the *Vssc* gene was undertaken to characterize DNA sequences from 300 *Ae*. *albopictus* at 6 specific codons: 989, 1011, 1016, 1532, 1534, and 1763 associated with pyrethroid resistance mutations in this species or in *Ae. aegypti*. Codon location and primer sequences are shown in Table 3.

**Table 3.** Codon location in the *Vssc* of *Aedes albopictus* and primer sequences used for Sanger sequencing

Domain	Exon	Codon	PCR primers	Reference	Primer sequence	Amplicon size (bp)	Sequencing primers	Reference	Primer sequence
I	10	410	aegSCF10	Tancredi et al. (2020)	GTGTTACGATCAGCTGGACC	162	aegSCF10	Tancredi et al. (2020)	GTGTTACGATCAGCTGGACC
			aegSCR10	Tancredi et al. (2020)	AAGCGCTTCTTCCTCGGC		aegSCR10	Tancredi et al. (2020)	AAGCGCTTCTTCCTCGGC
II	20	989	aegSCF20	Kasai et al. (2011)	GACAATGTGGATCGCTTCCC	480	aegSCF3	Kasai et al. (2011)	GTGGAACTTCACCGACTTCA
	20	1011	aegSCR21	Kasai et al. (2011)	GCAATCTGGCTTGTTAACTTG		aegSCR22	Kasai et al. (2011)	TTCACGAACTTGAGCGCGTTG
	21	1016							
III	29	1532	aegSCF7	Kasai et al. (2011)	GAGAACTCGCCGATGAACTT	740	Alb171F	Demok et al. (2019)	CCGATTCGCGAGACCAACAT
	29	1534	aegSCR7	Kasai et al. (2011)	GACGACGAAATCGAACAGGT		aegSCR8	Kasai et al. (2011)	TAGCTTTCAGCGGCTTCTTC
IV	?	1763	albSCF6	Kasai et al. (2011)	TCGAGAAGTACTTCGTGTCG	280	albSCF7	Kasai et al. (2011)	AGGTATCCGAACGTTGCTGT
			albSCR8	Kasai et al. (2011)	AACAGCAGGATCATGCTCTG				

A polymer chain reaction (PCR) master mix included final concentrations of Standard ThermoPol buffer Mg-free (1x) (New England Biolabs, Ipswich MA, USA), dNTPs (0.2 mM each) (Bioline, London UK), MgCl_2_ (1.5 mM) (Bioline, London, UK), 0.5 µM each of forward and reverse primers, 0.625 units of Immolase Taq polymerase (Bioline, London, UK), 2 µL genomic DNA (extracted with Qiagen DNeasy Blood and Tissue kit, Qiagen), and PCR-grade H_2_O to a final volume of 25 µL.

PCR conditions used to amplify the region were an initial denaturation of 95 °C for 10 min, 35 cycles of 95 °C for 30 s, annealment at 52 °C for 45 s, and extension at 72 °C for 45 s, followed by a final extension of 5 min at 72 °C and a hold at 10 °C. PCR amplicons were sent to Macrogen Inc. in Seoul, Korea, for purification and sequencing on a 3730xl DNA analyzer. PCR amplification was not always straightforward and multiple attempts were made to sequence individuals with missing data. Sequences were aligned and analyzed using Geneious 11.1.4 (Biomatters Ltd.) and mapped to reference sequences (Table 4).

**Table 4.** Reference sequences for *Aedes albopictus Vssc* domains I–IV

Domain	Reference	Accession no.	Genotype
I	Tancredi et al. (2020)	MN954411 MN954416 MN954417 MN954419	410[V] 410[A] 410[L] 410[G]
II	Kawada et al. (2020)	LC485547	989[S] 1011[I] 1016[V]
II	Zhou et al. (2019)	MK201606 MK201608	989[S] 1011[I] 1016[V] 989[S] 1011[I] 1016[G]
III	Zhou et al. (2019)	MK201619 MK201620	1532[T] 1534[F] 1532[I] 1534[F]
III	Zhu et al. (2019)	MN433726.1 MN433742.1 MN433752.1	1532[I] 1534[S] 1532[I] 1534[C] 1532[I] 1534[F]
IV	No reference sequences for *Aedesalbopictus* available. *Ae*. *aegypti* used as a reference: Chung et al. (2019)	MK495874.1 MK495875.1	1763[D] 1763[Y]

### Population Genomic Analysis

Further analysis of population genomic data generated during the study of Schmidt et al. (2020) was undertaken to identify single nucleotide polymorphisms (SNPs) that associate with *Vssc* genotypes of *Ae*. *albopictus*. Following methods described by Endersby-Harshman et al. (2020) for *Ae. aegypti*, a latent factor mixed model was used to identify SNPs across the genome that showed structuring in line with resistance allele genotypes compared with genome-wide structure. The latent factor mixed model was run in the R package LEA (Frichot and François 2015), using samples from the 3 populations where F1534C had been identified: Singapore, Timor-Leste, and Vanuatu. The model was first conditioned on genome-wide genetic structure present between the 3 populations, using *K* = 3 genetic clusters. For each individual, the number of copies of the F1534C alleles was used as the environmental variable. For the SNP dataset, the Stacks (Catchen et al. 2013) ref_map.pl workflow and program *populations* were used to build RADtag catalogs, call genotypes, and filter genotypes based on missing data (>95% global call rate, > 50% call rate in each population) and minor allele frequency (>0.05), leaving 114,817 SNPs. The model was run with 10 repetitions, 10,000 iterations, and a burn-in of 5,000.

Principal components analysis (PCA) was used to identify whether SNPs (outliers and non-outliers) showed structure indicative of the mutant genotypes having a common origin resulting from a single substitution event. PCAs were run in LEA, using either the entire dataset (114,817 SNPs) or only SNPs located within the *Vssc* gene region (39 SNPs).

We repeated the above analyses for 2 other mutations at the *Vssc* gene not known to confer resistance, which were also each found in 3 populations: 1016V, found in Malaysia, Singapore, and Vietnam; and 1763D, found in Thailand, Christmas Island, and Malaysia.

## Results

### Literature Review

Records of mutations in the *Vssc* gene of *Ae*. *albopictus* increased slowly after the initial discovery of F1534C in 2011 (Kasai et al. 2011). Another review of the field by Smith et al. (2016) only added F1534L which was found in *Ae*. *albopictus* from Florida, USA (Marcombe et al. 2014). However, by the time Moyes et al. (2017) completed another review a year later, F1534C had been found in China and Greece; F1534L had been reported from China and Italy; F1534S had appeared in China and the United States of America, and I1523T was discovered in Italy. Another review published in 2018 (Auteri et al. 2018) showed that mutations recorded in the *Vssc* gene of *Ae*. *albopictus* were still restricted to domain III.

In 2019, however, the V1016G mutation (domain II), common in *Ae*. *aegypti*, was reported in *Ae*. *albopictus* from Italy and Vietnam (samples collected in 2016) (Kasai et al. 2019) and from Beijing, China (samples collected in 2017 and 2017) (Zhou et al. 2019). Both studies used PCR primers (Kasai et al. 2011) to amplify partial sequences from domains II and III. Prior to these findings, other studies had used a similar approach to look for mutations in domain II, but had only found mutations in domain III: Marcombe et al. (2014) screened domains II, III, and IV in the United States of America; Xu et al. (2016) screened domains II, III, and IV in Asia, Africa, America, and Europe; Gao et al. (2018) screened domains II, III, and IV in Central, East, and South China; and Li et al. (2018) screened domains II, III, and IV in Guangzhou, China. A mutation in domain IV was also discovered in 2019, namely, D1763Y found by Lan et al. (2019) in *Ae*. *albopictus* from Yunnan province, China.

Despite the large rise in the number of mutations being reported from the *Vssc* of *Ae*. *albopictus*, the literature in 2022 and 2023 mostly focuses on survey data. The geographic distribution of the V1016G mutation was mapped across Europe by Pichler et al. (2022) who found it in 8 countries where it had not been found before. Twelve studies assessed associations between non-synonymous mutations and resistance levels identified in bioassays by comparing genotypes of dead and surviving mosquitoes (Table 5). Seven of the studies assessed the F1534S mutation and, in each case, an association with resistance to type II pyrethroids was demonstrated. One of the studies also showed an association of this mutation with type I pyrethroid resistance. F1534C, F1534L, and I1532T associations with type II pyrethroid resistance were not clearly demonstrated; associations were found in a small number of studies and not found in others (Table 5).

**Table 5.** Tests for association of *Vssc* mutations and resistance phenotypes in *Aedes albopictus*

Reference	Date	Location	Mutation	Test for resistance association	Association	No association
Balaska et al. (2020)	2020	Greece	F1534C, I1532T	Bioassays with deltamethrin—fully susceptible despite presence of mutant alleles.	None	F1534C, I1532T—type II
Chen et al. (2016)	2016	Haikou City, Hainan Island, China	F1534C/S/L (TGC/TCC/TTG)	Bioassay dead and survivors and χ^2^—correlation with F1534S and type II pyrethroid resistance (deltamethrin) and DDT resistance. Number of other mutations too low to be tested.	F1534S—type II	*
Gao et al. (2018)	2018	China	5.33% (I1532T), 44.20% (F1534S), 1.83% (F1534 L) and 0.87% (F1534C)	Odds Ratio F1534S association with permethrin and deltamethrin resistance. F1534 L/F1534C no association. Weak negative association for I1532T.	F1534S—types I and II	F1534 L, F1534C—types I and II. Weak negative association for I1532T.
Li et al. (2018)	2018	Southern China	F1534S, F1534L	Genotyped dead and survivors from bioassays. Odds ratio showed that both mutations conferred protection against deltamethrin (type II)	F1534S, F1534L—type II	*
Liu et al. (2020)	2020	Shandong, China	I1532T, F1534L/S	Tested dead and survivors from deltamethrin bioassay and used odds ratio. I1532T as heterozygote and F1534S were significantly correlated with deltamethrin survival. Odds ratio not significant for F1534L.	I1532T, F1534S—type II	F1534L
Modak and Saha (2022)	2022	West Bengal, India	F1534C, T1520I	Bioassays, but no correlation between mutation state and resistance phenotype for deltamethrin.	None	F1534C, T1520I—type II
Pichler et al. (2019)	2019	Italy, Albania, Greece	V1016G (Italy), F1534C (Greece), I1532T (Albania, Italy)	A significant association between 1016G and resistance to permethrin was observed (chi-squared = 154.02, *df* = 1, *P* < 0.0001). A significant association between 1534C with resistance to permethrin was observed (chi-squared = 15.11, *df* = 1, *P* < 0.0001). No association of 1532T with the resistant phenotype was observed (chi-squared = 1.25, *df* = 1, *P* = 0.26)	V1016G—type I, 1534C—type I	I1532T—type I
Rath et al. (2018)	2018	Odisha, India	F1534C	Significant correlation was detected between kdr mutations F1534C and DDT- or cyfluthrin-resistant phenotypes by chi-squared tests (*P* < 0.05).	F1534C—DDT and type II	*
Su et al. (2019)	2019	Guangzhou, China	F1534S, F1534L	Odds ratio shows F1534S and F1534L mutations significantly associated with resistance of *Ae*. *albopictus* to deltamethrin, permethrin and DDT (*P* < 0.05)	F1534S, F1534L—type I, type II, and DDT	*
Wei et al. (2021)	2021	China	F1534S/C/L, I1532T	Genotyping of dead and survivors. Association of 1534S and 1532T with deltamethrin survival. The association between the mutations and the resistance phenotype was verified by Fisher’s exact test or chi-squared test and the odds ratio (OR) was calculated for each mutation.	F1534S, I532T—type II	F1534C, F1534L—type II
Wu et al. (2021)	2021	China	F1534S, I1532T	Correlation analysis was conducted between the genotypes from codons 1532/1534 and the resistance phenotype in *Ae. albopictus*, indicating that the F1534S mutation was positively correlated with resistance phenotype to beta-cypermethrin (OR > 1, *P* < 0.05) and deltamethrin (OR > 1, *P* < 0.05), whereas the I1532T mutation was negatively correlated with resistance phenotype to beta-cypermethrin (OR < 1, *P* > 0.05), deltamethrin (OR < 1, *P* > 0.05), and permethrin (OR < 1, *P* > 0.05), although no statistical significance was found.	F1534S—type II	I1532T—type I, type II
Xu et al. (2016)	2016	Asia, Africa, America, Europe (12 locations)	China, Florida F1534S; China, Italy F1534L	To determine the association between *Vssc* mutations and resistance in two populations from southern China, Fisher’s exact test was performed and odds ratio was determined between resistant and susceptible mosquitoes for each *Vssc* allele. Two samples from southern China, WHO bioassay, deltamethrin—association for F1534S demonstrated clear association with resistant phenotype (odds ratio for Guangzhou 3.3, *P* < 0.0001; odds ratio for Shenzhen 2.7, *P* < 0.0001). F1534L mutation was not significantly associated with deltamethrin resistance in both populations (*P* > 0.05).	F1534S—type II	F1534L—type II

Three studies sought to elucidate the functional significance of the *Vssc* mutations in *Ae*. *albopictus* to determine mutation configurations causing pyrethroid resistance: Kasai et al. (2019) were able to establish homozygous lines for 1016G, 1534S, and 1534C as well as wild-type homozygotes of *Ae*. *albopictus*. Bioassays conducted on these lines demonstrated that the V1016G mutation led to much higher levels of resistance to both type I and type II pyrethroids than the mutations at codon 1534. A functional study using site-directed mutagenesis and electrophysiological assays in *Xenopus* oocytes (Yan et al. 2020) showed that mutations F1534S and F1534L conferred resistance to type I, but not type II pyrethroids. Most recently, the F1534S mutation has been verified as causing resistance to the type II pyrethroid, deltamethrin, in a study using CRISPR/Cas9 technology (Guo et al. 2022), contradicting findings of Yan et al. (2020).

**Fig. 1.**
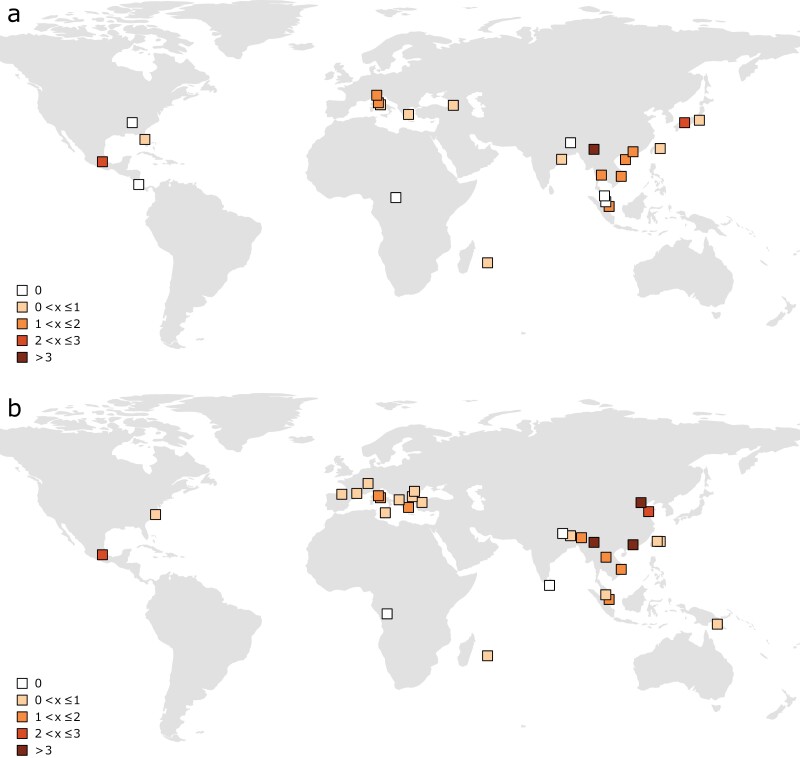
Number and location of all *Vssc* mutations recorded from a) 1994–2016 and b) 2016–2022, based on collection date of *Ae*. *albopictus*.

Based on the literature review and results from the current survey (see below), 26 amino acid changes have been recorded in the Vssc of *Ae*. *albopictus* across 26 countries (Table 1). The highest number of studies of the *Vssc* in *Ae*. *albopictus* have been conducted in China and the highest diversity in mutations is also found there. Two different substitutions have been shown to produce F1534S and 4 different substitutions can give rise to F1534L (Table 1) in *Ae*. *albopictus* from China. One of the variants of F1534L requires 2 substitutions from the wild-type state. Heterozygote 1534C/1534S individuals have also been found in China (Wei et al. 2021, Zheng et al. 2022, Yuan et al. 2023) along with 1534L/1534S (Liu et al. 2020, Wei et al. 2021) and the first report of phenylalanine to isoleucine mutation appearing as a heterozygote with 1534S (1534S/1534I) (Yuan et al. 2023). Italy is another focal point for studies in this area and 9 *Vssc* mutations have been recorded from *Ae*. *albopictus* in this country (Table 2). Overall, the distribution and number of *Vssc* mutations detected continue to increase (Fig. 1).

**Table 2.** *Aedes albopictus* worldwide distribution of *Vssc* mutations from the literature to date (2011–2023)—references to non-synonymous mutations by country

Laos	Vanuatu	Thailand	Mexico	Italy	Vietnam	La Réunion	China	Greece	Albania	Bulgaria*, France*, Georgia*, Malta*, Turkey*, Switzerland*, Spain*, Romania	USA	Timor Leste	PNG	India	Singapore	Malaysia	Indonesia
1 study	1	1	1	6	2	1	16	4	1	1	3	1	1	3	3	2	1
1 mutation	1	2	3	9	3	1	15	2	1	1	2	1	1	3	1	1	1
T1520I^b^	F1534C_1^a^	V410L^c^	V410L^c^	V410L^c^	V1016G^f^	V1016G^c^	S1000Y^h^	I1532T^t^	I1532T^d^	V1016G^ab^	F1534L_2^v^	F1534C_1^a^	F1534C_1^w^	V1016G^z^	F1534C_1^a^^,^^f^^,^^aa^^,^^ag^	F1534L_1^e^	F1534L_1^a^
		S989P^c^	S989Y^c^	V410A^c^	F1534C^f^^,^^g^		V1016G^c^^,^^h^^,^^ac^^,^^ae^	F1534C_1^c^^,^^d^^,^^s^^,^^t^			F1534S_1^s^^,^^u^			T1520I^x^			
			F1534L_1^c^	V410G^c^	F1534S?^f^		I1532T^h^^,^^m^^,^^p^^,^^r^^,^^ae^^,af*^							F1534C_1^x’y,z^			
				S989Y^c^			F1534C_1^c^^,I,^^k^^,^^l^^,^^m^^,^^p^^,^^q^^,^^ac^^,^^ae^										
				I1011V^c^			F1534C_2^c^										
				V1016G^c^^,^^d^^,^^f^^,ab*,^^ad^			F1534C_1/F1534S_1^q^^,^^ac^^,^^ae^										
							F1534S_1/F1534I^ae^										
				V1016I^c^			F1534L_1^c^^,^^h^^,^^k^^,^^l^^,^^p^^,^^q^^,^^s^^,^^ac^^,^^af^										
				I1532T^d^^,^^f^^,^^s^			F1534L_2^c^^,^^i^^,^^l^^,^^o^^,ac*^										
				F1534L_1^s^			F1534L_3^l^^,^^m^^,q*^										
							F1534L_4^l^										
							F1534L_1/F1534S_1^af^										
							F1534L_1/F1534S_2^q^										
							F1534R^l^										
							F1534S_1^c^^,^^h^^,^^i^^,^^j^^,^^k^^,^^l^^,^^m^^,^^n^^,^^o^^,^^p^^,^^q^^,^^r^^,^^s^^,^^ac^^,^^ae^^,^^af^										
							F1534S_2^c^^,^^l^^,^^p^										
							F1534S_1/F1534S_2^q^										
							F1534W^c^^,^^l^										
							D1763Y^p^										

Novel mutations C749 (Japan), A2023T (Singapore, Vietnam, and Japan), and G2046E (Japan) found in *Ae*. *albopictus* using exon capture (Itokawa et al. 2019) have not been included due to space restrictions.

^a^Current study.

^b^ Marcombe et al. (2022).

^c^ Tancredi et al. (2020).

^d^ Pichler et al. (2019).

^e^ Ahmad et al. (2020).

^f^ Kasai et al. (2019).

^g^ Vu et al. (2020).

^h^ Zhou et al. (2019).

^i^ Zhu et al. (2019).

^j^ Yang et al. (2021).

^k^ Chen et al. (2016).

^l^ Chen et al. (2021).

^m^ Gao et al. (2018).

^n^ Guo et al. (2022).

^o^ Su et al. (2019).

^p^ Lan et al. (2019).

^q^ Wei et al. (2021).

^r^ Wu et al. (2021).

^s^ Xu et al. (2016).

^t^ Balaska et al. (2020).

^u^ Abernathy et al. (2022).

^v^ Marcombe et al. (2014).

^w^ Katusele et al. (2022).

^x^ Modak and Saha (2022).

^y^ Rath et al. (2018).

^z^ Soni et al. (2018).

^aa^ Kasai et al. (2011).

^ab^ Pichler et al. (2022).

^ac^ Zheng et al. (2022).

^ad^ Pichler et al. (2021).

^ae^ Yuan et al. (2023).

^af^ Liu et al. (2020).

^ag^ Itokawa et al. (2019).

Not all studies of *Ae*. *albopictus* in the literature have identified *Vssc* mutations. Despite domains II and III of the *Vssc* in *Ae*. *albopictus* having been screened from Costa Rica (Chaves et al. 2015), Malaysia (Ishak et al. 2015, AhbiRami et al. 2020), and Nepal (Kawada et al. 2020), no non-synonymous mutations were found. Two studies of *Ae*. *albopictus* from India looking at *Vssc* domains II, III, and IV (Kushwah et al. 2015, Chatterjee et al. 2018) also failed to discover non-synonymous mutations, as did a similar study from Sri Lanka (Nugapola et al. 2021). Studies considering only codon 1534 from Papua New Guinea (Demok et al. 2019), the Central African Republic (Ngoagouni et al. 2016), and Congo specifically (Kamgang et al. 2020) did not find a mutation in this codon.

### *Vssc* Screening Survey

Results of multi-country screening in the current study showed that 46 individuals out of 300 had a *Vssc* mutation (synonymous or non-synonymous) (Table 6). Mutations occurred in codons 1016, 1532, 1534, and 1763. Sequence data are presented in Supplementary Table S1. No mutations were observed at codons 410, 989, and 1011. Non-synonymous mutations at the target codons were found in mosquitoes from Guangzhou, China; Jakarta, Indonesia; Selangor, Singapore; Timor Leste; and Vanuatu. Mutations at codon 1016 were synonymous in 5 individuals from Malaysia (Selangor), one from Singapore and one from Vietnam. Two mosquitoes from Guangzhou, China, showed the V1016G mutation as heterozygotes. At codon 1532, one mosquito each from Indonesia, Japan, and Sri Lanka was heterozygous for a synonymous mutation.

**Table 6.** Genotypes of *Ae*. *albopictus* showing synonymous and non-synonymous mutations in *Vssc* at targeted codons screened in the current study (*missing data, aa = amino acid, bold font = mutant)

Location	Sample	410	aa	989	aa	1011		1016	aa	1532	aa	1534	aa	1763	aa
Chiang Mai, Thailand	A_H4	GTA	V	*	*	*	*	*	*	ATC	I	TTC	F	**GAY**	D/D*
Christmas Is, Aus	A_E5	GTA	V	TCC	S	ATA	I	GTA	V	*	*	*	*	**GAY**	D/D*
Christmas Is, Aus	A_G5	GTA	V	TCC	S	ATA	I	GTA	V	ATC	I	TTC	F	**GAT**	D*
Guangzhou, China	A_D8	GTA	V	TCC	S	ATA	I	GTA	V	ATC	I	**TCC**	**S**	GAC	D
Guangzhou, China	A_E8	GTA	V	TCC	S	ATA	I	**GKA**	**V/G**	ATC	I	**TYC**	**F/S**	GAC	D
Guangzhou, China	A_G8	GTA	V	TCC	S	ATA	I	**GKA**	**V/G**	*	*	*	*	*	*
Guangzhou, China	A_H8	GTA	V	TCC	S	ATA	I	GTA	V	ATC	I	**TTS**	**F/L**	GAC	D
Guangzhou, China	A_A9	GTA	V	TCC	S	ATA	I	GTA	V	ATC	I	**TYC**	**F/S**	GAC	D
Guangzhou, China	A_B9	GTA	V	TCC	S	ATA	I	GTA	V	ATC	I	**TYC**	**F/S**	GAC	D
Guangzhou, China	A_C9	GTA	V	TCC	S	ATA	I	GTA	V	ATC	I	**TYC**	**F/S**	GAC	D
Guangzhou, China	A_D9	GTA	V	TCC	S	ATA	I	GTA	V	ATC	I	**TYS**	**F/S**	GAC	D
Guangzhou, China	A_A10	GTA	V	TCC	S	ATA	I	GTA	V	ATC	I	**TYC**	**F/S**	GAC	D
Guangzhou, China	A_B10	GTA	V	TCC	S	ATA	I	GTA	V	ATC	I	**TYC**	**F/S**	GAC	D
Guangzhou, China	A_D10	GTA	V	TCC	S	ATA	I	GTA	V	ATC	I	**TTS**	**F/L**	GAC	D
Guangzhou, China	A_E10	GTA	V	TCC	S	ATA	I	GTA	V	ATC	I	**YTC**	**F/L**	GAC	D
Jakarta, Indonesia	A_A11	GTA	V	TCC	S	ATA	I	GTA	V	**ATY**	I/I*	**TTS**	**F/L**	GAC	D
Japan	B_B1	GTA	V	TCC	S	ATA	I	GTA	V	**ATY**	I/I*	TTC	F	GAC	D
Malaysia (Pahang)	B_G5	GTA	V	*	*	*	*	GTA	V	ATC	I	TTC	F	**GAY**	D/D*
Malaysia (Pahang)	B_B6	GTA	V	TCC	S	ATA	I	GTA	V	ATC	I	TTC	F	**GAY**	D/D*
Malaysia (Selangor)	B_E7	GTA	V	TCC	S	ATA	I	**GTR**	V/V*	ATC	I	TTC	F	GAC	D
Malaysia (Selangor)	B_F7	GTA	V	*	*	*	*	**GTR**	V/V*	ATC	I	TTC	F	*	*
Malaysia (Selangor)	B_G7	GTA	V	*	*	*	*	**GTR**	V/V*	ATC	I	TTC	F	GAC	D
Malaysia (Selangor)	B_F8	GTA	V	TCC	S	ATA	I	**GTR**	V/V*	ATC	I	TTC	F	GAC	D
Malaysia (Selangor)	B_G8	*	*	*	*	*	*	**GTR**	V/V*	ATC	I	TTC	F	GAC	D
Singapore	B_E10	GTA	V	TCC	S	*	*	*	*	ATC	I	**TGC**	**C**	GAC	D
Singapore	B_F10	GTA	V	TCC	S	ATA	I	GTA	V	ATC	I	**TKC**	**F/C**	GAC	D
Singapore	B_G10	GTA	V	TCC	S	ATA	I	GTA	V	ATC	I	**TKC**	**F/C**	GAC	D
Singapore	B_H10	GTA	V	TCC	S	ATA	I	GTA	V	ATC	I	**TGC**	**C**	GAC	D
Singapore	B_A11	GTA	V	TCC	S	ATA	I	GTA	V	ATC	I	**TGC**	**C**	GAC	D
Singapore	B_B11	GTA	V	TCC	S	ATA	I	GTA	V	ATC	I	**TKC**	**F/C**	GAC	D
Singapore	B_C11	GTA	V	TCC	S	ATA	I	GTA	V	ATC	I	**TGC**	**C**	GAC	D
Singapore	B_D11	*	*	TCC	S	ATA	I	**GTG**	V*	ATC	I	TTC	F	GAC	D
Singapore	B_F11	GTA	V	TCC	S	ATA	I	GTA	V	ATC	I	**TKC**	**F/C**	GAC	D
Singapore	B_G11	*	*	TCC	S	ATA	I	GTA	V	ATC	I	**TKC**	**F/C**	GAC	D
Singapore	B_H11	GTA	V	*	*	*	*	*	*	ATC	I	**TGC**	**C**	GAC	D
Singapore	B_A12	GTA	V	TCC	S	ATA	I	GTA	V	ATC	I	**TKC**	**F/C**	GAC	D
Singapore	B_B12	GTA	V	*	*	*	*	*	*	ATC	I	**TGC**	**C**	GAC	D
Sri Lanka	C_A2	GTA	V	TCC	S	ATA	I	GTA	V	**ATY**	I/I*	TTC	F	GAC	D
Timor Leste	C_E4	GTA	V	TCC	S	ATA	I	GTA	V	ATC	I	**TKC**	**F/C**	GAC	D
Timor Leste	C_B5	GTA	V	TCC	S	ATA	I	GTA	V	ATC	I	**TKC**	**F/C**	GAC	D
Timor Leste	C_C5	GTA	V	TCC	S	ATA	I	GTA	V	ATC	I	**TKC**	**F/C**	GAC	D
Vanuatu	C_G8	GTA	V	TCC	S	ATA	I	GTA	V	ATC	I	**TGC**	**C**	GAC	D
Vanuatu	C_B9	GTA	V	TCC	S	ATA	I	GTA	V	ATC	I	**TKC**	**F/C**	GAC	D
Vanuatu	C_F9	GTA	V	TCC	S	ATA	I	GTA	V	ATC	I	**TKC**	**F/C**	GAC	D
Vanuatu	C_G9	GTA	V	*	*	*	*	GTA	V	ATC	I	**TKC**	**F/C**	GAC	D
Vietnam	C_D11	*	*	TCC	S	ATA	I	**GTG**	V*	*	*	*	*	GAC	D

As expected for *Ae*. *albopictus*, codon 1534 was the site of most mutations (Table 6). Eleven mosquitoes from Guangzhou, China, showed a non-synonymous mutation at this codon in either homozygous or heterozygous form and 2 amino acid variations were noted: F1534S and F1534L. F1534L as a heterozygote was also observed in one mosquito from Jakarta, the first report of a *Vssc* mutation in *Ae*. *albopictus* from Indonesia. The F1534C mutation was present in 12 mosquitoes from Singapore (6 homozygotes and 6 heterozygotes). Three mosquitoes from Timor Leste also showed this mutation as heterozygotes. We also observed one homozygote 1534C in Vanuatu and 3 heterozygotes. No *Vssc* mutations or screening attempts have been reported in *Ae*. *albopictus* from Timor Leste and Vanuatu until this study.

Codon 1763 in domain IV showed only synonymous mutations and these occurred as heterozygotes in one individual from Chiang Mai, Thailand, 2 mosquitoes from Malaysia (Pahang), and one from Christmas Island (Table 6). Another mosquito from Christmas Island was homozygous for the synonymous mutation. Only 2 mosquitoes in our sample had multiple mutations: one from Guangzhou, China, had genotypes V1016G and F1534S and a mosquito from Jakarta, Indonesia, showed I1532I (synonymous) and F1534L.

### Population Genomic Analysis

**Fig. 2.**
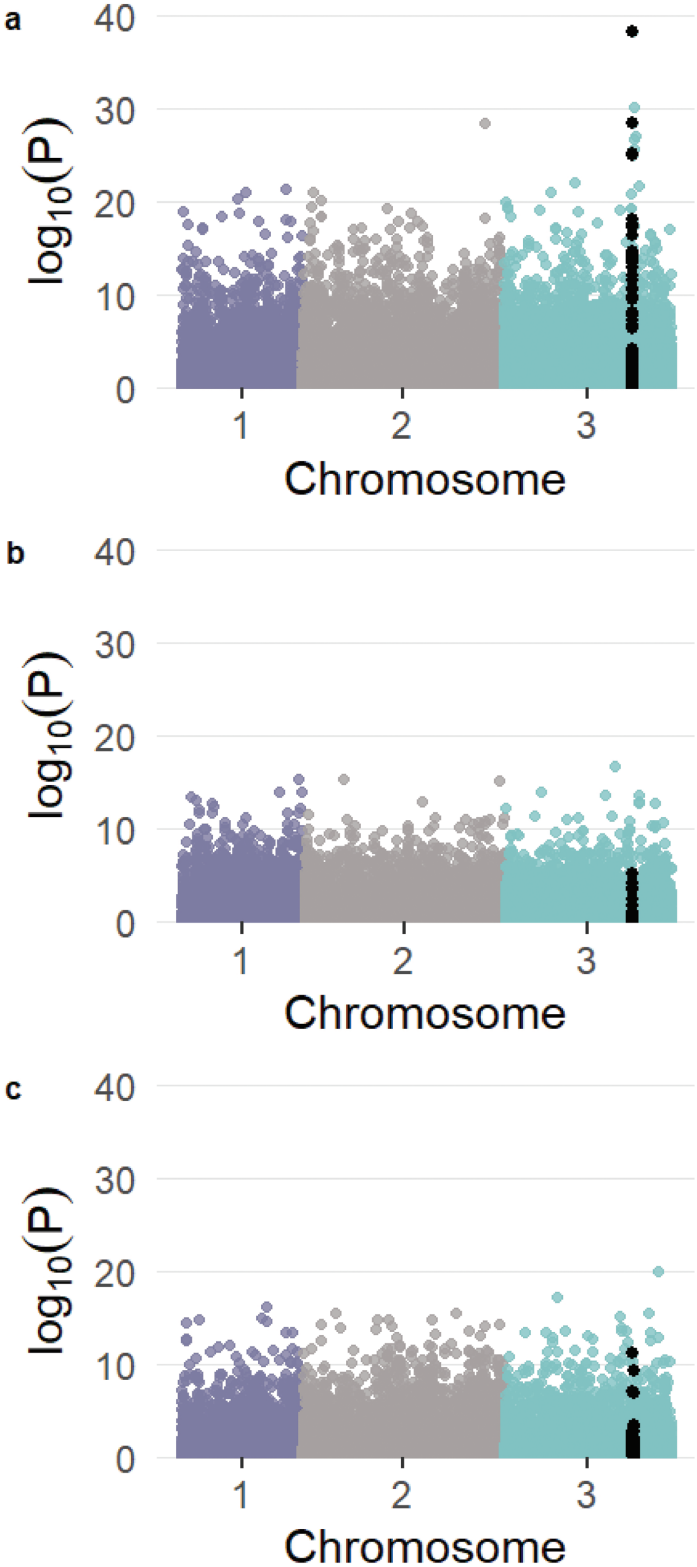
Manhattan plots of latent factor mixed models assessing association of genome-wide SNPs in *Ae*. *albopictus* to resistance genotype after conditioning on population structure. SNPs within 1Mb of the *Vssc* gene on chr3 are colored black. a) F1534C: Singapore, Timor Leste, Vanuatu; b) 1016V synonymous: Malaysia, Singapore, and Vietnam; and c) 1763D: Thailand, Christmas Island, and Malaysia.

**Fig. 3.**
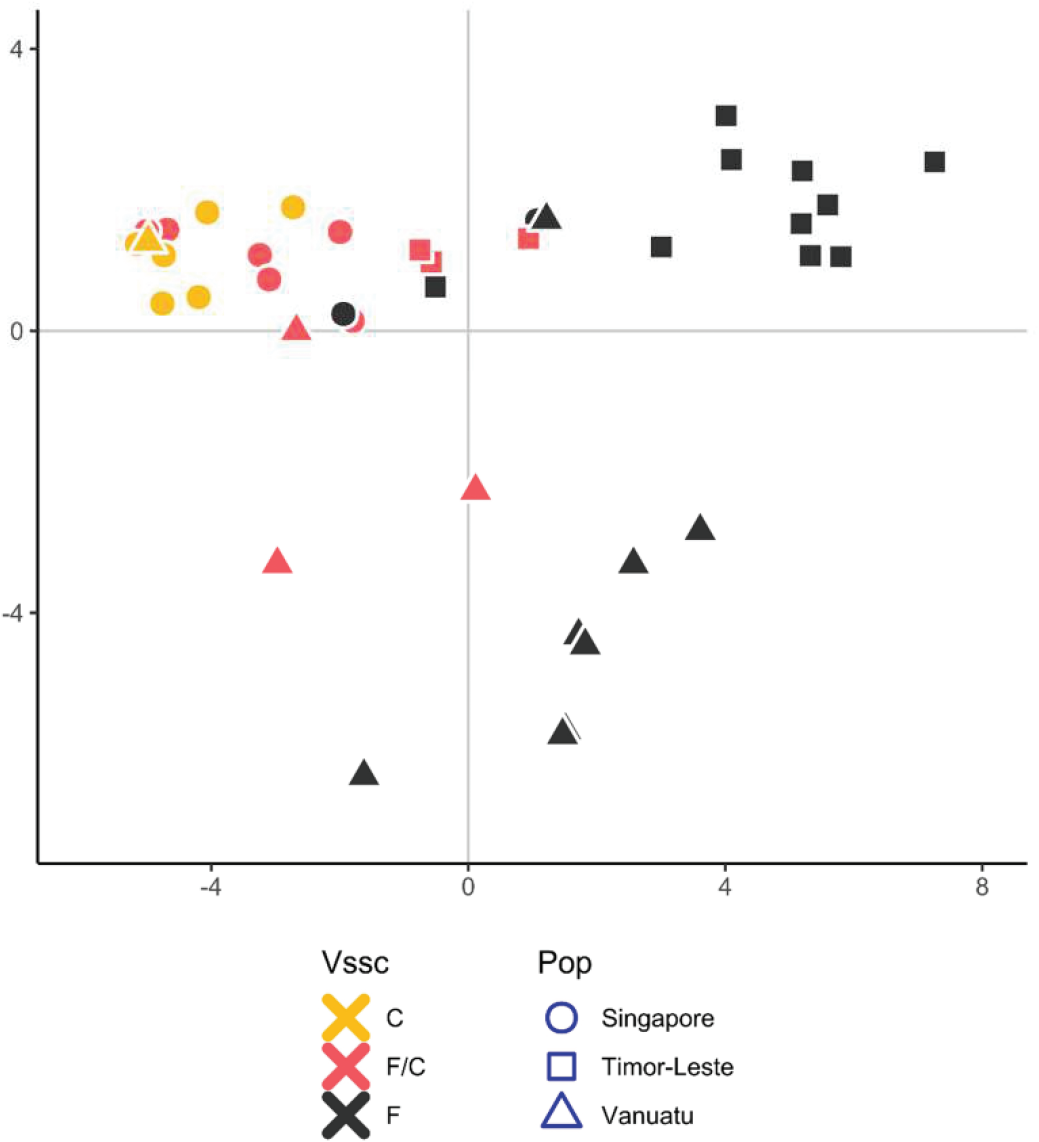
Principle components analysis of *Aedes albopictus*, using 39 SNPs located within the *Vssc* gene region. Points are color coded for *Vssc* genotype with F the wild type, C the homozygous resistant, and F/C the heterozygote. Shapes indicate sampling location (Singapore, Timor-Leste, and Vanuatu).

Latent factor mixed models indicated that, for the F1534C mutation, there was a peak of SNPs around the *Vssc* gene region that was strongly associated with F1534C genotype after conditioning for genetic structure among populations (Fig. 2a). There was no similar peak for either of the V1016 and D1763 synonymous mutations (Fig. 2b and c). Note that the genomic SNPs analyzed do not include those from the *Vssc* mutation sites (1016, 1534, and 1763) which do not appear in the ddRAD sequences, as only around 1–2% of the genome is assessed using this reduced representation genomic library sequencing method. PCA of the 39 SNPs within the *Vssc* gene showed structuring in line with F1534C genotype and not population of origin (Fig. 3). Individuals with F1534C alleles, and particularly homozygotes, separated from wild-type individuals along PC1. This clustering would be unexpected if the F1534C allele had arisen via multiple substitution events.

## Discussion

Different substitutions giving rise to the same amino acid change provide evidence for multiple origins of some of the *Vssc* genotypes of *Ae*. *albopictus*. Data gathered to date indicate that *Ae*. *albopictus* in China are under intense selection so that multiple *Vssc* mutations are becoming apparent with some genotypes reflecting more than one mutation at the same codon. Tancredi et al. (2020) emphasize that independent substitution events contribute more to resistance patterns than does gene flow in *Ae*. *albopictus*, using temporal information they gathered over 7 years. However, now that resistance alleles are increasing in frequency in some populations, the potential for resistance to spread, as invasive mosquitoes move around the globe, is heightened. Population genomic data combined with *Vssc* genotype analysis can be used to identify circumstances in which mutant alleles have arisen in other populations of the species by invasion and this information can be used to investigate incursion pathways.

Using population genomic data for *Ae*. *albopictus* individuals carrying the F1534C mutation in multiple locations, we were able to look for evidence of invasion of this mutation and found this situation for mosquitoes in Vanuatu and Timor Leste. The invasion could potentially be linked to Singapore, where the mutation was first discovered, given that the wild-type individuals (blue circles in Fig. 2) share the genomic background with the resistant individuals, whereas other wild types do not. However, increased sampling of wild-type individuals from Singapore and a larger sample size of locations overall would be needed to confirm this.

We observed selection on chromosome 3 close to the *Vssc* gene in *Ae*. *albopictus* from Singapore, Vanuatu, and Timor Leste in individuals that carried the non-synonymous mutation, F1534C (Fig. 2a). The same pattern was not observed for the synonymous mutations at codons 1016 and 1763 (Fig. 2b and c), which are not expected to be under selection. When a resistance profile becomes established via genetic invasion of individuals carrying a single de novo mutation, mutant individuals with identical resistance genotypes detected subsequently will have inherited these profiles from their invasive ancestors. They will have alleles that are identical by descent via this invasion which will be strongest among alleles at SNPs within and proximate to the *Vssc* gene region (chromosome 3), and, specifically, to the point mutation conferring resistance for that profile. We have observed similar patterns of variation at genomic SNPs within and around the *Vssc* gene region for individuals with the 1534C resistance profile from different populations (Singapore, Timor Leste, and Vanuatu). This is what we would expect from a resistance profile that had arrived at its current distribution via genetic invasion from a single source population.

Whether mutations arise via independent substitutions or are brought into populations by mosquito incursions, it is important to understand their impact when they are present. The global distribution of *Vssc* mutations in *Ae*. *albopictus* populations, although obfuscated by a concentration of studies in some regions such as China, shows that the V1016G and F1534C (TGC) mutations are recorded across the highest number of countries, whereas F1534S is proliferating in China (Table 1). Although incidence of *Vssc* mutations and pyrethroid resistance remains higher in *Ae*. *aegypti* than *Ae*. *albopictus*, the changes in mutation distribution and frequency since the discovery of F1534C in 2011 (mosquito collected in 2009) (Kasai et al. 2011), show that selection and probable dispersal of mutations is taking place. Extensive screening in Malaysia by Ishak et al. (2015) and AhbiRami et al. (2020) found no *Vssc* mutations in *Ae*. *albopictus*. However, Ahmad et al. (2020) found the F1534L mutation as a homozygote and 3 heterozygotes in a sample of 27 mosquitoes from Selangor state near Kuala Lumpur suggesting a recent advent of the mutation in this country. Similarly, no mutations at codon 1534 were found in *Ae*. *albopictus* in Papua New Guinea by Demok et al. (2019), but sampling in subsequent years revealed the F1534C mutation in 3 widely separated provinces (Katusele et al. 2022).

Moyes et al. (2017) stated that *Vssc* mutations may not be as strongly linked with pyrethroid resistance in *Ae*. *albopictus* as they are in *Ae*. *aegypti*. Most studies of *Ae*. *albopictus* show no association between F1534C and pyrethroid resistance (Table 5). Despite this, the demonstrated ability of this mutation to invade other populations suggests that it is being actively maintained. Most resistance association studies have been conducted since the 2017 statement of Moyes et al. (2017) and reveal a consistent pattern of association between the F1534S mutation and resistance to type I and type II pyrethroids at a significant level. Functional studies have confirmed a causative function of this allele to pyrethroid resistance (Yan et al. 2020, Guo et al. 2022). The detection of F1534S (TCC) in China, the United States, and possibly Vietnam is of concern, suggesting that this resistant genotype (Chen et al. 2016, Xu et al. 2016, Gao et al. 2018, Li et al. 2018, Wei et al. 2021, Wu et al. 2021, Guo et al. 2022, Yuan et al. 2023) is already spreading. A recent study by Chen et al. (2021) included historical samples and revealed the presence of the F1534S mutation (TCC) in *Ae*. *albopictus* in 1994. Genomic data could be used to compare general genomic backgrounds to see if this longstanding mutation has moved from China to these other countries. Similar studies could be conducted on the V1016G mutation which has also been shown to confer high levels of pyrethroid resistance in *Ae*. *albopictus* (Kasai et al. 2019, Pichler et al. 2019) and is becoming highly prevalent throughout multiple locations in Europe (Pichler et al. 2022).

Synonymous mutations have been identified in V1016, I1532, and F1534 from China (Chen et al. 2016, Zhou et al. 2019, Zheng et al. 2022, Yuan et al. 2023) and Italy (Pichler et al. 2021), coding for the same amino acid as in wild-type mosquitoes. We identified the same phenomenon for V1016 from Malaysia, Singapore, and Vietnam. D1763 from Thailand, Malaysia, and Christmas Island also showed synonymous mutations to the wild-type amino acid in our study. While synonymous mutations have not been considered as important in studies of insecticide resistance, recent discoveries in mammalian biology have shown that synonymy can affect the conformation, expressions, and function of proteins (Sauna and Kimchi-Sarfaty 2011). It may be of some importance to include synonymous mutations in future studies of insecticide resistance in *Ae*. *albopictus* and other pest species.

In summary, there is an increasing diversity of *Vssc* mutations being recorded in surveys of *Ae. albopictus* populations. Although there are relatively few attempts to link these mutations to resistance, with studies often indicating unclear relationships, there does seem to be a pattern of strong selection following invasion affecting the most common mutation identified so far. This finding raises issues around ongoing control options for *Ae. albopictus* based on pyrethroid insecticides, and emphasizes the importance of maintaining quarantine, even in cases where *Ae. albopictus* populations have become newly established, to limit the likelihood of genetic invasion. Given the importance of shipping in moving *Ae*. *albopictus* mosquitoes, at least in the Asia-Pacific region (Schmidt et al. 2020), it may be worth ensuring that a variety of control options are validated and used against incursions at points of entry.

## Supplementary material

Supplementary material is available at *Journal of Medical Entomology* online.

tjae005_suppl_Supplementary_Table_S1
