# Supplementary material for: Diversity and distribution of sodium channel mutations in Aedes albopictus (Diptera: Culicidae)
Source: J Med Entomol. 2024 Feb 16;61(3):630–43. doi: 10.1093/jme/tjae005 (PMC11078580; doi:10.1093/jme/tjae005)
Supplement: tjae005_suppl_Supplementary_Table_S1 [file tjae005_suppl_supplementary_table_s1.docx]

**Supplementary Data**

Table S1 – Sanger sequence data for *Aedes* *albopictus* voltage-sensitive sodium channel Domains II to IV – individuals with mutations (synonymous or non-synonymous)

| **Domain** | **Sequence data** |
| --- | --- |
| II | >AIIE8_aegSCR22 [organism=Aedes albopictus] Voltage-sensitive sodium channel Domain II Guangzhou  GKACTTAACCTTTTCTTAGCCTTGCTTTTGTCCAATTTCGGTTCATCGTCGCTGTCGGCACCGACGGCCGACAACGAAACGAACAAGATCGCGGAGGCGTTCAACCGGATA  >AIIG8_aegSCR22 [organism=Aedes albopictus] Voltage-sensitive sodium channel Domain II Guangzhou  GKACTTAACCTTTTCTTAGCCTTGCTTTTGTCCAATTTCGGTTCATCGTCGCTGTCGGCACCGACGGCCGACAACGAAACGAACAAGATCGCGGAGGCGTTCAACCGGATA  >BIIE7_aegSCF3 [organism=Aedes albopictus] Voltage-sensitive sodium channel Domain II Malaysia Selangor  GTRCTTAACCTTTTCTTAGCCTTGCTTTTGTCCAATTTCGGTTCATCGTCGCTRTCGGCACCGACGGCCGACAACGAAACGAACAAGATCGCGGAGGCGTTCAACCGGATATCGCGCTTCTCCAACTGGATCAAGTCCAACATCGCCAACGCGCTCAAGTTCGTGAAAAACAAGTTA  >BIID11__aegSCR22 [organism=Aedes albopictus] Voltage-sensitive sodium channel Domain II Singapore  GTGCTTAACCTTTTCTTAGCCTTGCTTTTGTCCAATTTCGGTTCATCGTCGCTATCGGCACCGACGGCCGACAACGAAACGAACAAGATCGCGGAGGCGTTCAACCGGA  >BIIF7_aegSCR22 [organism=Aedes albopictus] Voltage-sensitive sodium channel Domain II Malaysia Selangor  GTRCTTAACCTTTTCTTAGCCTTGCTTTTGTCCAATTTCGGTTCATCGTCGCTRTCGGCACCGACGGCCGACAACGAAACGAACAAGATCGCGGAGGCGTTCAACCGGATATCGCG  >BIIF8_aegSCR22 [organism=Aedes albopictus] Voltage-sensitive sodium channel Domain II Malaysia Selangor  GTRCTTAACCTTTTCTTAGCCTTGCTTTTGTCCAATTTCGGTTCATCGTCGCTRTCGGCACCGACGGCCGACAACGAAACGAACAAGATCGCGGAGGCGTTCAACCGGA  >BIIG7_aegSCR22 [organism=Aedes albopictus] Voltage-sensitive sodium channel Domain II Malaysia Selangor  GTRCTTAACCTTTTCTTAGCCTTGCTTTTGTCCAATTTCGGTTCATCGTCGCTRTCGGCACCGACGGCCGACAACGAAACGAACAAGATCGCGGAGGCGTCAACCGGA  >BIIG8_aegSCR22 [organism=Aedes albopictus] Voltage-sensitive sodium channel Domain II Malaysia Selangor  GTRCTTAACCTTTTCTTAGCCTTGCTTTTGTCCAATTTCGGTTYATCGTCGCTRTCGGCACCGACGGCCGACAACGAAACGAACAAGATCGCGGAGGCGTTCAACCGGATATCGCGCTCT  >CIID11_aegSCR22 [organism=Aedes albopictus] Voltage-sensitive sodium channel Domain II Vietnam  GTGCTTAACCTTTTCTTAGCCTTGCTTTTGTCCAATTTCGGTTCATCGTCGCTATCGGCACCGACGGCCGACAACGAAACGAACAAGATCGCGGAGGCGTCAACCGGATATCGCG |
| III | >AIIIA9_aegSCR8 [organism=Aedes albopictus] Voltage-sensitive sodium channel Domain III Guangzhou China  GTGGGCAAGCAGCCRATYCGCGAGACCAACATCTACATGTACCTCTACTTYGTGTTCTTCATCATCTYCGGGTCGTTCTTCACCCTYAAYCTGTTCATCGGTGTCATCATCGACAACTTCAACGAGCAGAAGAAGAAAGCCGGTGGCTCGCTGGAAATGTTCATGACGGAGGATCAGAAAAAGTACTACAAC  >AIIIA10_aegSCR8 [organism=Aedes albopictus] Voltage-sensitive sodium channel Domain III Guangzhou China  GTGGGCAAGCAGCCRATYCGCGAGACCAACATCTACATGTACCTCTACTTYGTGTTCTTCATCATCTYCGGGTCGTTCTTCACCCTYAAYCTGTTCATCGGTGTCATCATCGACAACTTCAACGAGCAGAAGAAGAAAGCCGGTGGCTCGCTGGAAATGTTCATGACGGAGGATCAGAAAAA  >AIIIA11_aegSCR8 [organism=Aedes albopictus] Voltage-sensitive sodium channel Domain III Jakarta Indonesia  GTGGGCAAGCAGCCGATTCGCGAGACCAACATYTACATGTACCTYTACTTYGTGTTCTTCATYATCTTSGGGTCGTTCTTCACCCTYMAYCTGTTYATCGGTGTCATCATYGACAAYTTCAACGAGCAGAAGAAGAAAGCCGGTGGCTCGCTGGAAATGTTCATGACGGAGGATCAGAAAAAGTACT  >AIIIB9_aegSCR8 [organism=Aedes albopictus] Voltage-sensitive sodium channel Domain III Guangzhou China  GTGGGCAAGCAGCCRATTCGCGAGACCAACATCTACATGTACCTCTACTTCGTGTTCTTCATCATCTYCGGGTCGTTCTTCACCCTYAAYCTGTTCATCGGTGTCATCATCGACAACTTCAACGAGCAGAAGAAGAAAGCCGGTGGCTCGCTGGAAATGTTCATGACGGAGGATCAGAAAAAGTACTACAACGC  >AIIIB10_aegSCR8 [organism=Aedes albopictus] Voltage-sensitive sodium channel Domain III Guangzhou China  GTGGGCAAGCAGCCRATTCGCGAGACCAACATCTACATGTACCTCTACTTCGTGTTCTTCATCATCTYCGGGTCGTTCTTCACCCTYMAYCYGTTCATCGGTGTCATCATCGACAACTTCAACGAGCAGAAGAAGAAAGCCGGTGGCTCGCTGGAAATGTTCATGACGGAGGATCAGAAAAAGTACTACAACG  >AIIIC9_aegSCR8 [organism=Aedes albopictus] Voltage-sensitive sodium channel Domain III Guangzhou China  GTGGGCAAGCAGCCRATTCGCGAGACCAACATCTACATGTACCTCTACTTYGTGTTCTTCATCATCTYCGGGTCGTTCTTCACCCTYAAYCTGTTCATCGGTGTCATCATCGACAACTTCAACGAGCAGAAGAAGAAAGCCGGTGGCTCGCTGGAAATGTTCATGACGGAGGATCAGAAAAAGTAC  >AIIID8_aegSCR8 [organism=Aedes albopictus] Voltage-sensitive sodium channel Domain III Guangzhou China  GTGGGCAAGCAGCCAATTCGCGAGACCAACATCTACATGTACCTCTACTTCGTGTTCTTCATCATCTCCGGGTCGTTCTTCACCCTTAATCTGTTCATCGGTGTCATCATCGACAACTTCAACGAGCAGAAGAAGAAAGCCGGTGGCTCGCTGGAAATGTTCATGACGGAGGATCAGAAAAAGTACTACAACG  >AIIID9_aegSCR8 [organism=Aedes albopictus] Voltage-sensitive sodium channel Domain III Guangzhou China  GTGGGCAAGCAGCCRATTCGCGAGACCAACATCTACATGTACCTCTACTTCGTGTTCTTCATCATCTYSGGGTCGTTCTTCACCCTYAAYCTGTTCATCGGTGTCATCATCGACAACTTCAACGAGCAGAAGAAGAAAGCCGGTGGCTCGCTGGAAATGTTCATGACGGAGGATCAGAAAAA  >AIIID10_aegSCR8 [organism=Aedes albopictus] Voltage-sensitive sodium channel Domain III Guangzhou China  GTGGGCAAGCAGCCGATTCGCGAGACCAACATCTACATGTACCTCTACTTYGTGTTCTTCATCATCTTSGGGTCGTTCTTCACCCTCAACCTGTTCATCGGTGTCATCATCGACAACTTCAACGAGCAGAAGAAGAAAGCCGGTGGCTCGCTGGAAATGTTCATGACGGAGGATCAGAAAAA  >AIIIE8_aegSCR8 [organism=Aedes albopictus] Voltage-sensitive sodium channel Domain III Guangzhou China  GTGGGCAAGCAGCCRATTCGCGAGACCAACATCTACATGTACCTCTACTTCGTGTTCTTCATCATCTYCGGGTCGTTCTTCACCCTYAAYCTGTTCATCGGTGTCATCATCGACAACTTCAACGAGCAGAAGAAGAAAGCCGGTGGCTCGCTGGAAATGTTCATGACGGAGGATCAGAAAAAGTACTACAACGC  >AIIIE10_aegSCR8 [organism=Aedes albopictus] Voltage-sensitive sodium channel Domain III Guangzhou China  GTGGGCAAGCAGCCRATTCGCGAGACCAACATCTACATGTACCTCTACTTCGTGTTCTTCATCATCYTCGGGTCGTTCTTCACCCTYAAYCTGTTCATCGGTGTCATCATCGACAACTTCAACGAGCAGAAGAAGAAAGCCGGTGGCTCGCTGGAAATGTTCATGACGGAGGATCAGAAAAA  >AIIIH8_aegSCR8 [organism=Aedes albopictus] Voltage-sensitive sodium channel Domain III Guangzhou China  GTGGGCAAGCAGCCGATYCGCGAGACCAACATCTACATGTACCTCTACTTYGTGTTCTTCATCATCTTSGGGTCGTTCTTCACCCTCAACCTGTTCATCGGTGTCATCATCGACAACTTCAACGAGCAGAAGAAGAAAGCCGGTGGCTCGCTGGAAATGTTCATGACGGAGGATCAGAAAAAGTACTACAACG  >BIIIA11_aegSCR8 [organism=Aedes albopictus] Voltage-sensitive sodium channel Domain III Singapore  GTGGGCAAGCAGCCGATCCGCGAGACCAACATCTACATGTACCTCTACTTTGTGTTCTTCATCATCTGCGGGTCGTTCTTCACCCTCAACCTGTTCATCGGTGTCATCATCGACAACTTCAACGAGCAGAAGAAGAAAGCCGGTGGCTCGCTGGAAATGTTCATGACGGAGGATCAGAAAA  >BIIIA12_aegSCR8 [organism=Aedes albopictus] Voltage-sensitive sodium channel Domain III Singapore  GTGGGCAAGCAGCCGATYCGCGAGACCAACATCTACATGTACCTCTACTTYGTGTTCTTCATCATCTKCGGGTCGTTCTTCACCCTCAACCTGTTCATCGGTGTCATCATCGACAACTTCAACGAGCAGAAGAAGAAAGCCGGTGGCTCGCTGGAAATGTTCATGACGGAGGATCAGAAAAA  >BIIIB11_aegSCR8 [organism=Aedes albopictus] Voltage-sensitive sodium channel Domain III Singapore  GTGGGCAAGCAGCCAATTCGCGAGACCAACATCTACATGTACCTCTACTTYGTGTTCTTCATCATCTKCGGGTCGTTCTTCACCCTCAACCTGTTCATCGGTGTCATCATCGACAACTTCAACGAGCAGAAGAAGAAAGCCGGTGGCTCGCTGGAAATGTTCATGACGGAGGATCAGAAAAAGTACTACAAC  >BIIIB12_aegSCR8 [organism=Aedes albopictus] Voltage-sensitive sodium channel Domain III Singapore  GTGGGCAAGCAGCCGATCCGCGAGACCAACATCTACATGTACCTCTACTTTGTGTTCTTCATCATCTGCGGGTCGTTCTTCACCCTCAACCTGTTCATCGGTGTCATCATCGACAACTTCAACGAGCAGAAGAAGAAAGCCGGTGGCTCGCTGGAAATGTTCATGACGGAGGATCAGAAAAA  >BIIIC11_aegSCR8 [organism=Aedes albopictus] Voltage-sensitive sodium channel Domain III Singapore  GTGGGCAAGCAGCCGATCCGCGAGACCAACATCTACATGTACCTCTACTTTGTGTTCTTCATCATCTGCGGGTCGTTCTTCACCCTCAACCTGTTCATCGGTGTCATCATCGACAACTTCAACGAGCAGAAGAAGAAAGCCGGTGGCTCGCTGGAAATGTTCATGACGGAGGATCAGAAAA  >BIIIE10_aegSCR8 [organism=Aedes albopictus] Voltage-sensitive sodium channel Domain III Singapore  GTGGGCAAGCAGCCGATCCGCGAGACCAACATCTACATGTACCTCTACTTTGTGTTCTTCATCATCTGCGGGTCGTTCTTCACCCTCAACCTGTTCATCGGTGTCATCATCGACAACTTCAACGAGCAGAAGAAGAAAGCCGGTGGCTCGCTGGAAATGTTCATGACGGAGGATCAGAAAA  >BIIIF10_aegSCR8 [organism=Aedes albopictus] Voltage-sensitive sodium channel Domain III Singapore  GTGGGCAAGCAGCCGATYCGCGAGACCAACATCTACATGTACCTCTACTTTGTGTTCTTCATCATCTKCGGGTCGTTCTTCACCCTCAACCTGTTCATCGGTGTCATCATCGACAACTTCAACGAGCAGAAGAAGAAAGCCGGTGGCTCGCTGGAAATGTTCATGACGGAGGATCAGAAAAA  >BIIIF11_aegSCR8 [organism=Aedes albopictus] Voltage-sensitive sodium channel Domain III Singapore  GTGGGCAAGCAGCCGATYCGCGAGACCAACATCTACATGTACCTCTACTTTGTGTTCTTCATCATCTKCGGGTCGTTCTTCACCCTCAACCTGTTCATCGGTGTCATCATYGACAACTTCAACGAGCAGAAGAAGAAAGCCGGTGGCTCGCTGGAAATGTTCATGACGGAGGATCAGAAAAA  >BIIIG10_aegSCR8 [organism=Aedes albopictus] Voltage-sensitive sodium channel Domain III Singapore  GTGGGCAAGCAGCCGATYCGCGAGACCAACATCTACATGTACCTCTACTTYGTGTTCTTCATCATCTKCGGGTCGTTCTTCACCCTCAACCTGTTCATCGGTGTCATCATCGACAACTTCAACGAGCAGAAGAAGAAAGCCGGTGGCTCGCTGGAAATGTTCATGACGGAGGATCAGAAAAA  >BIIIG11_aegSCR8 [organism=Aedes albopictus] Voltage-sensitive sodium channel Domain III Singapore  GTGGGCAAGCAGCCGATYCGCGAGACCAACATCTACATGTACCTCTACTTYGTGTTCTTCATCATCTKCGGGTCGTTCTTCACCCTCAACCTGTTCATCGGTGTCATCATCGACAACTTCAACGAGCAGAAGAAGAAAGCCGGTGGCTCGCTGGAAATGTTCATGACGGAGGATCAGAAAAAGTAC  >BIIIH10_aegSCR8 [organism=Aedes albopictus] Voltage-sensitive sodium channel Domain III Singapore  GTGGGCAAGCAGCCGATCCGCGAGACCAACATCTACATGTACCTCTACTTTGTGTTCTTCATCATCTGCGGGTCGTTCTTCACCCTCAACCTGTTCATCGGTGTCATCATCGACAACTTCAACGAGCAGAAGAAGAAAGCCGGTGGCTCGCTGGAAATGTTCATGACGGAGGATCAGAAAAAGTACTACAACG  >BIIIH11_aegSCR8 [organism=Aedes albopictus] Voltage-sensitive sodium channel Domain III Singapore  GTGGGCAAGCAGCCGATCCGCGAGACCAACATCTACATGTACCTCTACTTTGTGTTCTTCATCATCTGCGGGTCGTTCTTCACCCTCAACCTGTTCATCGGTGTCATCATCGACAACTTCAACGAGCAGAAGAAGAAAGCCGGTGGCTCGCTGGAAATGTTCATGACGGAGGATCAGAAAAAGTACTACAACG  >BIIIB1_aegSCR8 [organism=Aedes albopictus] Voltage-sensitive sodium channel Domain III Japan  GTGGGCAAGCAGCCAATTCGCGAGACCAACATCTACATGTACCTCTACTTYGTGTTCTTCATYATCTTCGGGTCGTTCTTCAMSSTYMAYCTGTTYATCGGTGTCATCATCGACAACTT  >CIIIA2_aegSCR8 [organism=Aedes albopictus] Voltage-sensitive sodium channel Domain III Sri Lanka  GTGGGCAARCAGCCGATTCGCGAGACCAACATCTACATGTACCTCTACTTYGTGTTCTTCATYATCTTCGGGTCGTTCTTCACCCTCAACCTGTTCATCGGTGTCATCATCGACAACTTCAACGAGCAGAAGAAGAAAGCCGGTGGCTCGCTGGAAATGTTCATGACGGAGGATCAGAAAAAGTACTACAACG  >CIIIB5_aegSCR8 [organism=Aedes albopictus] Voltage-sensitive sodium channel Domain III Timor Leste  GTGGGCAAGCAGCCRATYCGCGAGACCAACATCTACATGTACCTCTACTTTGTGTTCTTCATCATCTKCGGGTCGTTCTTCACCCTCAACCTGTTCATCGGTGTCATCATCGACAACTTCAACGAGCAGAAGAAGAAAGCCGGTGGCTCGCTGGAAATGTTCATGACGGAGGATCAGAAAAAG  >CIIIB9_aegSCR8 [organism=Aedes albopictus] Voltage-sensitive sodium channel Domain III Vanuatu  GTGGGCAAGCAGCCGATCCGCGAGACCAACATCTACATGTACCTCTACTTTGTGTTCTTCATCATCTKCGGGTCGTTCTTCACCCTCAACCTGTTCATCGGTGTCATCATCGACAACTTCAACGAGCAGAAGAAGAAAGCCGGTGGCTCGCTGGAAATGTTCATGACGGAGGATCAGAAAAAGTAC  >CIIIC5_aegSCR8 [organism=Aedes albopictus] Voltage-sensitive sodium channel Domain III Timor Leste  GTGGGCAAGCAGCCGATYCGCGAGACCAACATCTACATGTACCTCTACTTYGTGTTCTTCATCATCTKCGGGTCGTTCTTCACCCTYAAYCTGTTCATCGGTGTCATCATCGACAACTTCAACGAGCAGAAGAAGAAAGCCGGTGGCTCGCTGGAAATGTTCATGACGGAGGATCAGAAAAA  >CIIIE4_aegSCR8 [organism=Aedes albopictus] Voltage-sensitive sodium channel Domain III Timor Leste  CGAGACCAACATCTACATGTACCTCTACTTTGTGTTCTTCATCATCTKCGGGTCGTTCTTCACCCTCAAYCTGTTCATCGGTGTCATCATCGACAACTTCAACGAGCAGAAGAAGAAAGCCGGTGGCTCGCTGGAAATGTTCATGACGGAGGATCAGAAAAA  >CIIIF9_aegSCR8 [organism=Aedes albopictus] Voltage-sensitive sodium channel Domain III Vanuatu  GTGGGCAAGCAGCCGATCCGCGAGACCAACATCTACATGTACCTCTACTTTGTGTTCTTCATCATCTKCGGGTCGTTCTTCACCCTCAACCTGTTCATCGGTGTCATCATCGACAACTTCAACGAGCAGAAGAAGAAAGCCGGTGGCTCGCTGGAAATGTTCATGACGGAGGATCAGAAAAA  >CIIIG8_aegSCR8 [organism=Aedes albopictus] Voltage-sensitive sodium channel Domain III Vanuatu  GTGGGCAAGCAGCCGATCCGCGAGACCAACATCTACATGTACCTCTACTTTGTGTTCTTCATCATCTGCGGGTCGTTCTTCACCCTCAACCTGTTCATCGGTGTCATCATCGACAACTTCAACGAGCAGAAGAAGAAAGCCGGTGGCTCGCTGGAAATGTTCATGACGGAGGATCAGAAAAAGTACTACAACGC  >CIIIG9_aegSCR8 [organism=Aedes albopictus] Voltage-sensitive sodium channel Domain III Vanuatu  GTGGGCAAGCAGCCRATYCGCGAGACCAACATCTACATGTACCTCTACTTYGTGTTCTTCATCATCTKCGGGTCGTTCTTCACCCTCAACCTGTTCATCGGTGTCATCATCGACAACTTCAACGAGCAGAAGAAGAAAGCCGGTGGCTCGCTGGAAATGTTCATGACGGAGGATCAGAAAAA |
| IV | >AIVE5_albSCF7 [organism=Aedes albopictus] Voltage-sensitive sodium channel Domain IV Christmas Island Australia  TCTGTCTGCTGCTGTTCTTGGTGATGTTCATCTTYGCCATCTTCGGGATGTCGTTCTTCATGCACGTGAAGGAYAAGAGCGGGCTGGACGACGTGTACAAYTTYAAGACGTTCGGC  >AIVG5_albSCF7 [organism=Aedes albopictus] Voltage-sensitive sodium channel Domain IV Christmas Island Australia  TCTTGGTGATGTTCATCTTTGCCATCTTCGGGATGTCGTTCTTCATGCACGTGAAGGATAAGAGCGGGCTGGACGACGTGTACRATTTTAAGACGTTCGGC  >AIVH4_albSCF7 [organism=Aedes albopictus] Voltage-sensitive sodium channel Domain IV Thailand  TCTGTCTGCTGCTGTTCTTGGTGATGTTCATCTTYGCCATCTTCGGGATGTCGTTCTTCATGCACGTGAAGGAYAAGAGCGGGCTGGACGAYGTGTACRAYTTYAAGACGTTCGGC  >BIVB6_albSCF7 [organism=Aedes albopictus] Voltage-sensitive sodium channel Domain IV Malaysia Pahang  TCTGTCTGCTGCTGTTCTTGGTGATGTTCATCTTCGCCATCTTCGGGATGTCGTTCTTCATGCACGTGAAGGAYAAGAGCGGGCTGGAYGAYGTGTAYAATTTTAAGACGTTCGGC  >BIVG5_albSCF7 [organism=Aedes albopictus] Voltage-sensitive sodium channel Domain IV Malaysia Pahang  TCTGTCTGCTGCTGTTCTTGGTGATGTTCATCTTCGCCATCTTCGGGATGTCGTTCTTCATGCACGTGAAGGAYAAGAGCGGGCTGGACGACGTGTACAATTTYAAGACGTTCGGC |
